# What are the lived healthcare experiences of patients with primary sclerosing cholangitis? A community-based qualitative interview study

**DOI:** 10.1136/bmjopen-2023-082498

**Published:** 2025-02-06

**Authors:** Katherine Arndtz, Madeline Cameron, Gideon Hirschfield, Jayne Parry, Sheila Greenfield

**Affiliations:** 1 Institute of Immunology and Immunotherapy, University of Birmingham, Birmingham, UK; 2 Centre for Liver Research, NIHR Birmingham Liver Biomedical Research Unit, Birmingham, UK; 3 The Autoimmune and Rare Liver Disease Programme, Division of Gastroenterology and Hepatology, Toronto General Hospital, University Health Network, Toronto, Ontario, Canada; 4 Public Health, University of Birmingham, Birmingham, UK; 5 Institute of Applied Health Research, University of Birmingham, Birmingham, UK

**Keywords:** Patients, Hepatology, Primary Care, QUALITATIVE RESEARCH, Chronic Disease

## Abstract

**Abstract:**

**Objectives:**

Primary sclerosing cholangitis (PSC) is a rare chronic disease that presents challenges to both patients and clinicians. This study aimed to explore the experiences of patients with PSC regarding their disease and healthcare.

**Design:**

A series of semi-structured interviews was completed with patients with PSC, including questioning their experiences of living with PSC and its related healthcare.

**Setting:**

Participants were recruited from communities in England, Scotland and Wales, with advertisement via PSC Support (UK disease-specific charity support group).

**Participants:**

18 patients aged between 21 and 72 years were interviewed; 10 were male (56%), and all were of Caucasian ethnicity. Inclusion criteria were as follows: adults, self-identifying as having a diagnosis of PSC, and currently under National Health Service treatment for this disease. Patients with a history of liver transplantation were excluded.

**Results:**

Participants confirmed the ongoing physical and psychological burden of PSC and its related healthcare. Living with PSC was described as a journey; the timeline of events was important to patients, with particular challenges identified along the way. These included difficulties in obtaining a diagnosis and accessing timely and knowledgeable medical care. Overcoming the unusual combination of uncertainties that PSC presents was of particular concern to participants; these differ from those observed in more common chronic diseases with established treatment pathways. Hidden complexities within chronic illness behaviour in PSC were described, including a noteworthy fragile doctor−patient relationship and dependence on the specialist. These produce additional challenges for the optimal clinical management of such patients by generalists and specialists.

**Conclusions:**

This study complements the existing literature on the ongoing high burden of PSC, with added value from in-depth discussions with patients themselves. Priorities for further work have been identified, including the need for improved risk stratification tools to allow individualised management and prognostication, as well as improving access to knowledgeable care while maintaining a strong doctor–patient relationship.

**STRENGTHS AND LIMITATIONS OF THIS STUDY**

- In this study, in-depth interviews were performed to gain insight into the depth of the lived experiences of patients with Primary sclerosing cholangitis from across Great Britain and the spectrum of disease, irrespective of the basis of their clinical management.
- Real-life challenges faced by patients have been highlighted, resulting in practical ideas for change in both generalist and specialist clinical management that could improve the experience for not only patients with PSC but also those with other chronic diseases.
- Recruitment was through a single national charitable support group that was involved in the conception and design of the study.
- A mixture of interview mediums was used, and a lack of ethnic diversity was observed within the study, with the potential to introduce bias.

## Introduction

 Primary sclerosing cholangitis (PSC) is a chronic immune-mediated liver disease characterised by cholestasis and biliary stricturing, leading to cholangitis, cirrhosis and a high risk of hepatobiliary malignancy and liver transplantation^1^ . The incidence ranges from 0.91 to 1.3/100,000 populations, with people of Northern European descent more likely to be affected.^1^ The unpredictable prognosis and lack of disease-modifying therapy in PSC result in an unusual set of circumstances in which those affected must navigate.

Attempts have been made to assess the quality of life (QoL) in PSC using existing quantitative disease-scoring questionnaires or developing new scoring systems,^2^,^4^ with a 2016 study using additional free-text responses for more detailed analysis.^5^ These studies have found that patients with PSC have lower health-related QoL than healthy controls as well as a significant psychological burden, including social isolation and existential anxiety, on top of a heavy symptom burden. These studies primarily used quantitative questionnaire-based scoring systems rather than formal qualitative research methods, which would allow a deeper understanding of patients’ lived experiences.

A recent qualitative study explored the experiences of patients with PSC as a chronic disease, identifying three major themes of patient experience, including emotional distress related to an often invisible illness, psychological effects of coping with PSC as a chronic disease, and complexities behind self-management in an unpredictable condition.^6^ This study was based at a single specialist centre in Germany in which telephone interviews were performed and focused on how patients perceive their illness over time. Patients’ lived experiences across geographical regions and how these experiences relate to the specialist and non-specialist medical care they received remain understudied.

Appreciation of these patient experiences is relevant to patients and their supporters, as well as to their clinicians and other chronic disease cohorts, allowing for more person-oriented clinical management and the development of outcome measures of improved relevance to patients.

### Aims

This study aimed to explore the experiences of patients with PSC regarding their disease and its related UK healthcare. Specific objectives were to explore the following:

1. How was the diagnosis reached, and what is its impact on the individual?
2. What is the personal experience of living with PSC as a chronic disease?
3. What is the experience of patients receiving PSC-related UK healthcare?

## Results

### Subject demographics

25 individuals answered the screening questions, with 18 subsequently selected for interview. Screening questions included age, gender, ethnicity, geographical location, site of PSC clinician and severity of disease. For this study, the absence of cirrhosis, PSC-related hospital admission and liver transplant assessment were considered for the early disease stage, and this was self-reported by potential participants. See online supplemental file 1 for methods.^7^,^9^

Table 1 shows participant demographics; limited demographic information is given due to the rarity of PSC and the resulting potential risk of the participants being identified. 14 interviews were completed in person and 4 via telephone, ranging from 45 to 90 min in length. Participants were aged 20–80 years and were 2–16 years into their PSC diagnosis. 10 were male, and 11 had co-morbid inflammatory bowel disease (IBD). All interviewees were White British or European and were from across England, Scotland and Wales. No expressions of interest were received from other ethnic groups.

**Table 1:** Study population demographics

Study number	Gender	Age range (years)	Years since diagnosis	Location of PSC treatment	Assessment of disease severity	Symptoms present	IBD present
10	Male	50–60	2	1	Transplant assessment	Yes	Yes
11	Female	20–30	5	1	Stable cirrhosis	Yes	Yes
12	Male	30–40	5	1	Transplant assessment	Yes	Yes
13	Male	70–80	3	1	Recurrent cholangitis	Yes	No
14	Female	60–70	4	1	Early disease	Yes	No
15	Female	50–60	3	1	Early disease	Yes	No
16	Male	30–40	12	2	Transplant assessment	Yes	Yes
17	Female	60–70	14	3	Early disease	No	No
18	Female	50–60	16	3	Early disease	Yes	No
19	Female	50–60	9	4	Early disease	Yes	Yes
20	Male	40–50	6	5	Early disease	No	Yes
21	Female	50–60	2	6	Early disease	Yes	No
22	Male	30–40	5	7	Early disease	No	Yes
23	Male	50–60	25	8	Listed for transplant	Yes	No
24	Female	30–40	6	9	Early disease	Yes	Yes
25	Male	60–70	2	10	Early disease	Yes	Yes
26	Male	50–60	4	11	Recurrent cholangitis	Yes	Yes
27	Male	70–80	15	1	Recurrent cholangitis	Yes	Yes

IBDinflammatory bowel diseasePSCprimary sclerosing cholangitis

Females in this study had a less severe disease; one reported cirrhosis, and none described hospitalisation or transplant assessment. In contrast, seven males reported severe disease, including four who had undergone liver transplant assessment.

A summary of the nine main themes identified and their sub-themes is shown in table 2, along with the study numbers (as per table 1) in which interviews have different themes. The personal impact of PSC, including the uncertainty of the future, the possible need for liver transplantation and need for trustworthy information about personal trajectory emerged in all patient interviews.

**Table 2:** Summary of themes identified from patients with PSCinterviews

Theme	Subtheme	Interview number (total)
Before PSC	Baseline health status	11, 12, 15, 16, 17, 21 (6)
	Comorbid IBD	10, 11, 12, 16, 18, 19, 21, 22, 25, 26, 27 (11)
PSC symptoms	Pain	11, 12, 13, 14, 18, 21, 23, 24, 26, 27 (10)
	Itch	12, 13, 16, 18, 19, 20, 21, 24, 26, 27 (10)
	Fatigue	10, 11, 12, 13, 14, 15, 16, 18, 19, 20, 21, 23, 24, 25, 26, 27 (18)
	Cholangitis	10, 11, 12, 13, 14, 16, 18, 21, 23, 24, 25, 26 (12)
	Weight loss	10, 11, 12, 14, 16, 20, 22, 23, 27 (9)
	‘Brain fog’	12, 13, 14, 16, 19, 20, 21, 23, 24, 25, 26, 27 (12)
Diagnostic process	Complex process	10, 11, 12, 13, 14, 15, 20, 21, 23, 24, 25, 27 (12)
	Multiple hospitals	10, 12, 14, 18, 22, 23, 24, 25, 26, 27 (10)
	Incorrect diagnosis	10, 12, 14, 18, 22, 23, 24, 25, 26, 27 (9)
	Diagnosis moment	10, 11, 12, 13, 14, 16, 17, 18, 19, 20, 25, 26 (12)
	Effect on patients	11, 12, 13, 15, 16, 19, 20, 21, 23, 24, 25, 26, 27 (13)
PSC medical management	Treatment	ALL (18)
	Hospital admissions	10, 11, 12, 13, 16, 23, 26 (7)
	Research	10, 12, 15, 16, 17, 18, 19, 20, 21, 24, 26, 27 (12)
	Monitoring	10, 15, 16, 17, 18, 22, 23, 24, 25, 26, 27 (11)
Outcomes	Liver transplant	ALL (18)
	Cancer	10, 13, 17, 18, 19, 21, 23, 25, 27 (9)
	Death	10, 11, 12, 14, 15, 16, 17, 18, 19, 20, 22, 23, 24, 25, 26 (15)
Experience of healthcare	System failure	10, 11, 14, 16, 18, 20, 21, 23, 24, 25, 26 (11)
	Access	10, 11, 12, 14, 16, 17, 20, 23, 24, 25, 26, 27 (12)
	Importance of the specialist	10, 13, 14, 15, 16, 17, 18, 21, 24, 26, 27 (11)
	Doctor–patient relationship	10,11, 12, 13, 14, 15, 16, 17, 22, 24, 25, 26, 27 (13)
Personal effect of the disease	Disrupted narrative	ALL (18))
	Stigma/isolation	10, 12, 13, 14, 15, 17, 18, 20, 22, 24, 25, 26 (12)
	Social impact	10, 11, 12, 14, 15, 16, 17, 18, 19, 20, 21, 22, 23, 24, 25, 26, 27 (17)
	Lucky	14, 15, 17, 18, 19, 20, 23, 24, 26, 27 (10)
	Low mood	11, 12, 16, 17, 19, 21, 23, 24, 26, 27 (10)
Importance of information	Seeking knowledge	All (18)
	Peer support/self-advocating	10, 11, 12, 14, 15, 16, 17, 18, 19, 20, 21, 22, 23, 24, 25, 26, 27 (17)
	The expert patient	10, 11, 12, 14, 15, 16, 18, 21, 22, 23, 25, 26, 27 (13)
	Exchange between providers	12, 13, 14, 16, 17, 18, 24, 25, 26, 27 (10)
Uncertainty	Prognosis	ALL (18)
	Day-to-day	10, 13, 14, 19, 20, 21, 23, 24, 26, 27 (10)
	Treatment	13, 17, 18, 19, 22 (5)

IBDinflammatory bowel diseasePSCprimary sclerosing cholangitis

**Figure 1:**
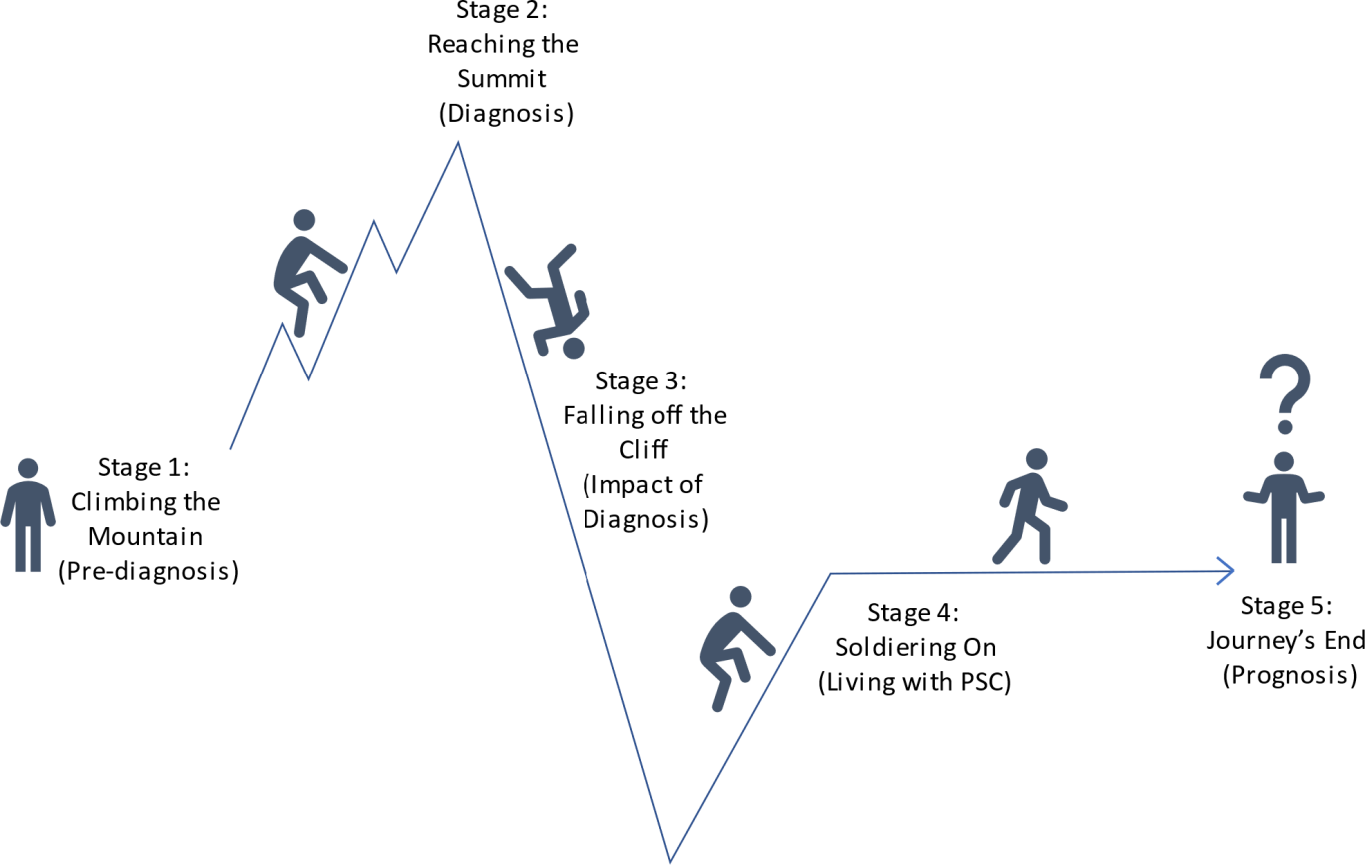
Journey of patients with PSC. A pictorial representation of key stages in the journey for people living with PSC. PSC, primary sclerosing cholangitis.

Reflection on these initial themes identified that all patients had described their experiences as one long difficult journey, an accepted approach to qualitative analysis.^10^ Five common stages to this journey were identified; therefore, these themes were similarly reordered, as discussed below and shown in figure 1. Exemplar quotes are used to demonstrate the themes identified.

### Stage 1: climbing the mountain (prediagnosis stage)

#### Symptoms

Most participants were symptomatic at the beginning of their PSC journey (n = 16), the severity of which tended to progress over time; two participants remained asymptomatic for a long period. The most common symptoms were fatigue, cholangitis, brain ‘fog’, abdominal pain and itching (table 3). Most participants described a combination of multiple symptoms.

**Table 3:** PSC symptoms as experienced by patients

Symptom	Quotes
Abdominal pain	*I just get like pain in my, my liver…like a knitting needle* (018)*I could barely walk, this pain was so bad* (026)
Brain fog	*It’s sort of just slowly feels like knowledge is ebbing out…of my brain* (024)
	*I do get this horrible brain fog and it’s very negative…you’re drunk but you haven’t had any alcohol…I’m not a half full person when I’m in that state. I’m really half empty* (023)
Cholangitis	*Basically feeling terrible…aching everywhere, rigors* (026)
	*They pumped me full of antibiotics and pain relief and God knows what else to try and get the infection under control…I was constantly in and out with infections* (011)
Fatigue	*It’s like a blanket coming over you and I just can’t keep my eyes open* (026)
	*Tired all the time…someone had pulled the plug and energy was just going down the plughole* (023)
	*Felt like I was walking through treacle* (025)
Itching	*Itching absolutely drove me insane…nothing really got on top of it…its unbearable* (026)
	*I’ve ripped my skin to bits, I’ve got scars all over my body…I go to work and I’ve got scabs all over my face* (016)
Weight loss	*I’d stopped going on the scales after losing 30 pounds* (027)
	*I’d lost loads of weight, I think I weighed 38 kilos* (011)
Multiple symptoms	*Jelly legs, wooliness in the head…extreme tiredness…lack of appetite…twinges in the side or back, pain in the top of my right shoulder…nausea* (013)

PSCprimary sclerosing cholangitis

All symptomatic participants experienced fatigue, describing this as an all-encompassing weakness of their body and mind, which left them unable to carry out normal daily activities. The resulting inability to work full time, or at all, was commonly described. Abdominal pain was frequently described, which could be both physically and mentally disabling, and itching was particularly distressing on personal and social levels. Rigors and sweating were common initial symptoms of cholangitis, often associated with worsening of other symptoms including itch, abdominal pain and fatigue. The unpredictability of these episodes was of particular concern to the participants.

#### Diagnostic process

For most participants, the diagnostic process was complex. The exception was those with IBD, who tended to be diagnosed quicker, often on routine blood test monitoring. Half of the cohort described being investigated for, or initially diagnosed with, alternative conditions; ultimately, incorrect diagnoses included alcohol excess, mental illness, occupational exposure, atypical infection, irritable bowel syndrome and growing pains. Weight loss commonly initiated investigations to exclude cancer. Three participants described the reassurance they were given once cancer was ruled out; however, they were subsequently discharged, without a diagnosis being made, and subsequently perceived the disease as being ‘dismissed’ (023).

The time from the onset of symptoms/investigations to the diagnosis ranged between 3 and 9 years, which was deemed unacceptable by most participants.

I knew that something was wrong…no one was really interested in my story, in helping me. (012)

#### Loss of faith in healthcare

Frustration was clearly demonstrated within the interviews; 12 participants described how they were seen by multiple doctors in different settings, how little progress they felt was made and how they were ‘starting from scratch’ (027) each time. Conflicts with clinicians were common at this stage. Three participants described searching for answers elsewhere and using private healthcare, dietary intervention and/or complementary therapies. A loss of faith in the National Health Service (NHS) and medicine was observed in which interviewees perceived a failure of their clinicians to believe that they had a physical ailment and manage it correctly:

I totally lost faith…to gastroenterologists because they thought they knew, and they didn’t. And, and they weren’t honest enough to say that they didn’t know. (027)

### Stage 2: reaching the summit (diagnosis event)

#### Relief of a diagnosis

Receiving the diagnosis was a major life event for all participants. Most felt better able to cope after receiving the diagnosis of PSC, and three participants described their initial relief to receive a diagnosis; the unknown was deemed to be more frightening. However, this relief was usually short-lived as participants found out more about PSC and its complications.

It seemed like such a relief after such a long time to know that I wasn't mad and there actually was something wrong… I thought ‘well this is the answer to all the problems, surely in this day and age there isn't going to be an illness that you can't treat’…naively. (021)

Two-thirds of the participants were highly critical of how their clinician broke the news of their diagnosis, with one describing this as being handled ‘appallingly badly’ (019). Participants with milder clinical disease appeared more psychologically affected by their diagnosis than those with pre-existing co-morbidities. Discovering the lack of treatment and its potential need for transplantation was a shock for all.

I wish I didn’t know…a condition that there’s no treatment for, what’s the point to knowing you’ve got it because it’s not going to make any difference. (017)

### Stage 3: falling off the cliff (aftermath of the diagnosis)

#### Search for information

Immediately postdiagnosis, all but one participant began to search for more information. Few patients had been signposted towards any specific sources or patient support groups; those who had were all diagnosed by a hepatologist in a tertiary centre. The information participants found themselves was universally disheartening, and some felt that such an undirected search should be actively discouraged by clinicians.

I don’t trust Google because you could have 100 people with PSC that are doing fine…two people who are having a bad time of it and struggling…They’re the only two you’re going to read about. (010)

Having a clinician able to personalise information and make it relevant to their personal trajectory was of great importance to participants.

#### Existential crisis

Most participants described an initial depression after receiving their diagnosis. Many became convinced that they were going to die soon, especially the female participants. Grief was commonly described as they came to terms with a life different from that which they had envisioned. Clinical depression was commonly diagnosed following the PSC diagnosis; participants directly related this to having PSC, rather than it being an independent mental illness. Anger was also commonly observed, whether at the unfairness of the situation, given they had led a healthy lifestyle, or due to the lack of treatment options.

I’m the perfect, healthy person. I never drank. I never smoked…I did everything that I thought was possible to keep myself healthy and… it didn’t make any difference, did it? (017)

However, three participants described how they had made positive changes in their outlook since being diagnosed, vowing to live each day to the fullest.

#### Public perceptions and stigma

The first thing many interviewees did after being diagnosed was to tell family and friends. The rarity of PSC, poor general knowledge about liver disease overall and the often-hidden nature of their symptoms were all cited as factors limiting the ability of others to appreciate what participants were experiencing. Over half of the participants described worrying that others, including some clinicians, thought they must be responsible for their illness, with alcohol excess being a common misperception.

If you told somebody you’d had a heart attack, or a stroke, or you’d got Parkinson’s disease, then the reaction always is, ‘Aww’ but when you have a liver disease, people make judgments and think it’s a lifestyle choice. (017)

### Stage 4: soldiering on (living with chronic primary sclerosing cholangitis)

10 participants went to multiple hospitals for their PSC, usually a local non-specialist unit and a regional liver centre. The rest were solely managed by a transplant centre, with one participant exclusively monitored in their local district general hospital. Many described stark differences in their experiences by comparing local and specialist centres, especially in terms of the improved understanding they gained from their specialist appointments and more intensive management of their symptoms and their IBD. The pill burden was significant for many.

Monitoring was acknowledged to be important by two-thirds of the interviewees, all of whom were troubled by the unpredictable nature of PSC. Participants were aware of the controversies surrounding the use of ursodeoxycholic acid, variations in management between centres and the overall lack of other disease-modifying treatments. This introduced additional uncertainty for some participants that they might miss out on more optimal healthcare elsewhere; this added additional strain to their relationship with their clinicians, and most were keen to remain under a PSC specialist for the long term.

I want to know that I’m being looked after properly…I would like to be under a more specialist hospital…I’m just worried that I’ll miss out. (016)

#### System failure

The management of PSC as a chronic illness was described as challenging. This included the often poor communication between hospitals, multiple appointments for different specialties and participants’ need to generally advocate for themselves to ensure they were not overlooked. The lack of continuity and consistency of care was a concern for nearly half of the participants, who described how receiving conflicting information further undermined their trust in the system and their clinicians. Having direct access to their specialist was deemed helpful by all, and many ended up only trusting information directly from their specialist centre.

If they’re in a different hospital they have a different way of doing it…you end you end up not really trusting anything unless you’re sat in front of your hepatologist. (018)

#### Doctor–patient relationship

Many participants described long-running tensions with their medical team. The lack of treatment was frustrating for most; they felt that their doctors should be able to offer more, even though they knew from their research that this was not possible.

If you’re a patient you can’t tell the difference between a doctor not having the answers because there aren’t any and a doctor not having the answers because they just don’t know them. (018)

A successful doctor–patient relationship appeared inherently based on effective information exchange; where participants felt that they were listened to and received information in a language that they could understand, their relationship with their medical team became stronger.

#### Emergency access to care

Nearly half of the interviewees described repeated PSC-related hospital admissions. Cholangitis was common, and accessing emergency care was a frequent but usually frustrating event for many. Conflict with emergency clinicians was commonly described, with participants knowing it was their PSC that was causing them to be ill but that the clinicians did not agree, instead treating them for other things or confusing PSC with other conditions, such as primary biliary cholangitis. This further undermined their trust in clinicians and the healthcare system.

All participants found that their social life significantly deteriorated because of PSC. Isolation was commonly described, worsened by PSC being so rare a disease.

I think it’s quite isolating…if you have breast cancer you’d know somebody or you’d know of somebody who knew somebody. So you’d be able to find somebody that you could talk to. (017)

Participants also described a gradual loss of support from their loved ones, including the breakdown of family relationships due to this lack of a diagnosis, which was subsequently restored once PSC was confirmed.

When I got the diagnosis, I think everyone was relieved because I think everyone could finally understand what had been going on. (023)

Due to the study being recruited via PSC support, all participants were already engaged with this group and other patient groups, such as Liver North. These were deemed to be safe spaces for patients to ask questions and compare their medical care. However, some participants found it distressing to see into their potential future by meeting those severely affected by the disease.

### Stage 5: where the road leads (future outcome)

From the moment of diagnosis, all participants described being in the knowledge that they would eventually deteriorate. Despite this, 11 participants described stable PSC, without complications such as liver failure, cholangitis or need for transplant. Four participants had previously undergone transplant assessment (three being currently active on the transplant list), with an additional three experiencing regular cholangitis requiring hospital admissions.

All participants broached the possibility of liver transplantation within their interviews; most felt that this was a lifeline, and the idea of not being eligible for this was unimaginable, given the poor quality (and quantity) of a future without one. Many found the idea of transplant distressing and the uncertainties while waiting on the list even more so. The guilt for needing someone to die so they could benefit and the grief for their future donor were commonly described, as was the duty of care that came with such a ‘gift*’ *(010).

I want my life back…I feel like I’m in a draw, like there’s so many people with PSC that are similar to me, more advanced than me, and they’ve not had transplants. (016)

#### Uncertainty

Above any specific worries about their prognosis, the inherent unpredictability and uncertainty of PSC profoundly affected all the participants. Not knowing how or when they might progress or when symptoms would hit was distressing. Fluctuating symptoms were particularly hard to cope with, as every small change led to anxieties of further deterioration. Many described not feeling able to plan for tomorrow, let alone the future, impacting all aspects of their social, family and work lives.

So apart from the actually physical, medical aspect of the PSC, which has been horrendous at times…the unpredictability of it is one of the worst things…it’s like flicking a switch. (026)

#### Acceptance

Half of the participants described developing a level of acceptance over time, especially observed in those with more severe disease. 10 participants described feeling ‘lucky’ that they had access to good medical care or transplant, or due to having milder disease. Taking control was important to participants, as was making positive adjustments to their outlook.

I’m a very much a ‘choosing life’ person…I want to have the best experience that I can within the constraints of what I’ve got. (023)

#### How experiences can be improved

**Figure 2:**
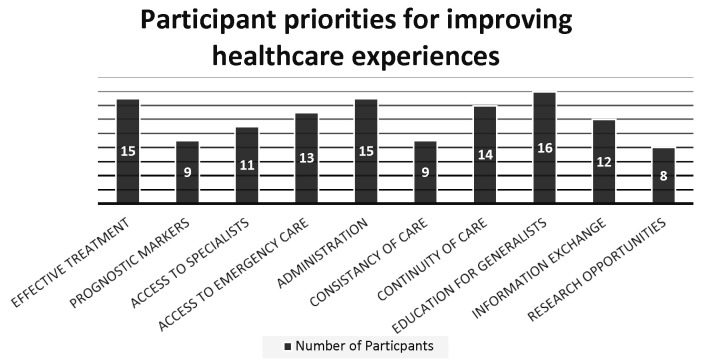
Priorities of interviewees for improving future healthcare. The priorities identified by participants for improving future healthcare for people living with primary sclerosing cholangitis.

All interviewees felt strongly that their medical management could be improved (figure 2). The most common requests were the discovery of new treatments, improving access to knowledgeable medical care (both via generalists and specialists), better continuity and consistency of care and improved coordination between departments and specialties. Patients also wanted access to more detailed information about the progression of their liver disease and what trajectory they were personally facing.

## Discussion

This study explored patients with PSC experiences of their disease, healthcare management and how these might practically be improved. Themes already identified in the literature^6^ in this same disease cohort were confirmed; the importance of patient experience, psychological effects of coping with a chronic illness and difficulty in patients self-managing an unpredictable disease were all common themes. This study confirmed these themes in the UK PSC population, suggesting a more universal patient experience not reliant on geography, culture or local healthcare systems.

The study has identified the importance of the timeline, with participants seeing their experiences as a long and arduous journey. Multi-faceted uncertainty was a prominent theme, with many being deeply affected by the insecurity of where their journey would end. The lifetime patient burden of PSC has been confirmed, with a high frequency and variability of physical symptoms and psychological consequences. Fatigue was the most debilitating symptom experienced by interviewees, likely under-appreciated by clinicians.

Patient impacts of healthcare interventions for PSC have not been explored in depth previously in the literature. Understanding these is vital to optimally manage these complex patients and those with other chronic illnesses. PSC, however, represents particular challenges to those affected, given its rarity, unpredictable prognosis and lack of disease-modifying treatments. This study adds further depth to patient experiences of their disease-related healthcare in both specialist and more local centres, which has not previously been explored. The UK’s NHS model of healthcare is not representative of healthcare systems in all other countries; this study has also highlighted the particular difficulties UK patients face. Participants struggled to access optimal medical management and navigating complex administrative inflexibilities across multiple healthcare providers was frustrating for them.

Various issues of importance to patients, which were identified in this study, reflect their acceptance of chronic illness behaviour. Difficulties described in managing medical regimes, disrupted biography and the importance of information have all been well described in the literature.^11^ The fundamental need for legitimisation of the presence of illness was key and lack of this left participants unable to access the traditional sick role.^12^ Grief for lost health was observed, as was the resulting ‘biographical disruption’^13^ often leading to an ‘existential crisis’.^14^ The health paradox was observed,^15^ that is, previously healthy interviewees appeared more psychologically affected by their PSC diagnosis than those with significant pre-existing illness. Female interviewees had objectively less severe liver disease yet subjectively a lower self-perceived QoL; compared with men, women report stronger feelings of vulnerability to illness, greater felt stress and an overall different perception of their health^16^, with further effects from the disproportionate domestic burden carried mainly by women, even in modern times.^17^

However, the striking theme throughout the interviews was uncertainty, which was the most distressing aspect of PSC. Interviewees described a perfect storm of prediagnostic, trajectory and symptomatic uncertainty.^11^ Although described in other chronic diseases, the lack of validated disease-modifying treatments, variations in monitoring strategies and difficulties in accessing knowledgeable medical care for PSC all enhanced the personal effect of these uncertainties. Our data complement similar findings from Loesken *et al*, who described PSC as a ‘wave-like experience’.^6^ Therefore, it is not surprising that patients with PSC carry a huge psychological burden, which likely affects their health-seeking behaviour, need for information and reliance on reassurance from a doctor they trust. A resulting strong dependence on specialist input was observed, and the importance of a strong doctor–patient relationship in this cohort cannot be underestimated.

The study has highlighted practical improvements in clinical management, which are important to patients and are achievable now, and research is ongoing for effective treatment and monitoring strategies. Signposting patients to access approved sources of information is feasible and requires no new infrastructure, and peer-reviewed pamphlets are freely available online, for example, from the British Liver Trust and PSC Support. Robust referral systems into and out of specialist care are necessary to ensure that the most appropriate patients are seen promptly and patients are assured that they are receiving the best possible management. Telemedicine may have a role in standardising the quality of care across geographical barriers and is increasingly used for routine clinical care in a postpandemic world. However, this must be balanced by the potential impacts virtual consultations may have on the fragile doctor−patient relationships identified in this study. Given the unpredictability of PSC, specialist helpline access, such as those successfully run nationally for other conditions, would allow for more responsive management when patients need it most. Additionally, patient-held personalised ‘passports’ may aid non-specialist medical staff in providing appropriate and timely treatment, especially in emergency situations.

## supplementary material

10.1136/bmjopen-2023-082498 online supplemental file 1

## Data Availability

Data are available upon reasonable request.
