# Supplementary material for: What are the lived healthcare experiences of patients with primary sclerosing cholangitis? A community-based qualitative interview study
Source: BMJ Open. 2025 Feb 6;15(2):e082498. doi: 10.1136/bmjopen-2023-082498 (PMC11800207; doi:10.1136/bmjopen-2023-082498)
Supplement: online supplemental file 1 [file bmjopen-15-2-s001.docx]

Supplemental Material – K Arndtz October 30 Submission

# Methods

Qualitative research uses open-ended data gathered from personal interactions, which is subsequently collated to present the richness of ideas and breadth of experiences. This allows for an understanding of the complexities of how people experience the world, their illnesses and their healthcare, that quantitative data cannot provide. This study used semi-structured interviews(9) with UK adults with PSC. This allowed discussion of several pre-determined topics within all interviews including diagnosis, medical management and living with chronic illness. Participants were also asked their key priorities for future changes to their healthcare. It was felt important by the research team to ensure that the research methods and approach should reflect the patient voice throughout the study therefore an inductive approach to analysis was used to allow themes to freely emerge, and analysis was concurrent with data collection, the “constant-comparative” method (10-1).

*Study design & sampling*

A purposive sampling technique9 was employed to gather as broad an experience of PSC as possible. The study was advertised via PSC Support, a UK-based charitable support group. After applicants expressed interest in the study, a small dataset was created including geographic, demographic and disease severity data. Participants were subsequently selected for interview to provide maximum diversity of these characteristics. To reduce bias, analysis was independently verified by JP and SG (medical sociologists).

*Study population*

Pre-transplant patients with PSC were selected, allowing for more detailed exploration of PSC without the inevitable physical and psychological complications undergoing transplantation would introduce into the findings.

Inclusion criteria were:

1. Patients over the age of 18
2. Able and willing to give informed consent
3. Who self-reported they had a diagnosis of PSC
4. Who had experienced UK healthcare for their PSC

Participants with a previous liver transplant were excluded.

*Data Collection*

Interviews were carried out between 15/12/2017 and 21/5/2018 by a sole interviewer (KA), a female doctor who had undergone training in qualitative research. Interviews were completed using a Topic Guide (see Supplementary material) which was created in collaboration with PSC Support patient representatives. The main areas covered were diagnosis, medical management and living with chronic illness. Interviews were face-to-face where possible; telephone interviews were permitted to broaden the recruitment potential. Open questioning was used to encourage participants tell their stories, and to demonstrate their personal trajectory and healthcare experiences. Interviews were recorded and transcribed verbatim prior to analysis; transcription was via a professional transcription service with a confidentiality agreement in place.

Researcher Characteristics and *Patient & Public Involvement*

The lead researcher was a female doctor who had clinical experience in managing patients with PSC in a tertiary care setting. Any potential influential impact of the researcher on the study set up or results was reduced by the researcher undergoing specialist qualitative research methods training and by involving patient participants in the research from the initial concept stage. Patient representatives from PSC Support were involved in the creation of the study design and were especially vital in the creation of the interview Topic Guide to ensure the data collected during the interviews was of maximum relevance to patients themselves. .

*Interview analysis*

Data was analysed thematically[10](#_bookmark12) with data collection simultaneous with analysis, known as the constant-comparative method[11](#_bookmark13). Thus the analysis continuously evolved as the dataset

expanded. Coding was done by hand, with the researcher scanning transcripts for experiences highlighted by interviewees to be of particular importance to them. Therese were then refined after discussion with a team of experienced qualitative researchers into common themes. Analysis was continually refined until no new themes were identified. This “conceptual saturation”(7) is predicted to be reached within 15-20 interviews (8), provided the sample is selected to give maximum variability. This occurred after 18 interviews at which point data collection ceased.

*Ethical Considerations & Funding*

Ethical approval was via the University of Birmingham Ethical Review Committee (ERN_16-0130); NHS ethical approval was not required. Funding for participant travel expenses and transcribing was via charitable donations. Patient confidentiality was maintained throughout. Unique study numbers were used on all documentation, which was stored on a secure university server or locked filing cabinet on university premises. All data will be destroyed after 10 years, as per the University of Birmingham’s Code of Conduct for Research[12](#_bookmark14). Informed written consent was gained from all interviewees.
