# Supplementary material for: What are the lived healthcare experiences of patients with primary sclerosing cholangitis? A community-based qualitative interview study
Source: BMJ Open. 2025 Feb 6;15(2):e082498. doi: 10.1136/bmjopen-2023-082498 (PMC11800207; doi:10.1136/bmjopen-2023-082498)
Supplement: Supplementary file 2 [file bmjopen-2023-082498.reviewer_comments.pdf]

PEER REVIEW HISTORY

BMJ Open publishes all reviews undertaken for accepted manuscripts. Reviewers are asked to complete a checklist review form and are provided with free text boxes to elaborate on their assessment. These free text comments are reproduced below.

ARTICLE DETAILS

Title (Provisional)

What are the lived healthcare experiences of patients with primary sclerosing cholangitis? a qualitative interview study

Authors

Arndtz, Katherine; Cameron, Madeline; Hirschfield, Gideon; Parry, Jayne; Greenfield, Sheila

---

VERSION 1 - REVIEW

**Reviewer**	**1**
**Name**	**Ranieri, Veronica**
**Affiliation**	**Tavistock and Portman NHS Foundation Trust, Research & Development**
**Date**	**20-Dec-2023**
**COI**	**None**

Thank you for the opportunity to read and review the article. I suggest the article be accepted subject to minor revisions.

It is disheartening to read that the life experiences and interactions of individuals living with PSC with healthcare services have remained this way – I say this as I was part of a qualitative study looking at the experiences of those living with PSC and their caregivers from a psychological standpoint (doi:e0234624). Upon looking at the authors' period of data collection, it is possible that there may have been overlap between our samples as we both recruited from advertisements via PSC Support potentially up to a year apart. I imagine that it is all our hopes that, ten years from now, these narratives may change.

The study adds some new perspectives on the experiences of individuals living with PSC, for instance on public perceptions and stigma. The addition of stages is also a helpful way of describing the experiences of diagnosis onwards.

I would invite the authors to make the following changes:

• Though there are fewer published studies on the lived experiences and psychological impact of PSC (of which the authors describe in their results) in comparison to those

chronicling the physical symptoms and medical management of the illness, I would suggest that the authors provide a more ample introduction and comparison to previous literature within the discussion on what is already known and how their study's findings differ.

• 'The end of the road' may reflect the anxiety and hopelessness some individuals feel as they think of or approach advanced liver illness. I wonder, however, if this could be revised as am mindful that some individuals with PSC may read this and feel worse.

• I would be cautious about including the statement that "women can have stronger feelings of vulnerability to illness, greater felt stress and an overall different perception of their own health when compared to men". This may or may not be true, depending on whether you believe that women experience these difficulties more or are just more likely to report these.

Reviewer **2**

Name **Toussaint, Anne**

Affiliation **University Medical Center Hamburg-Eppendorf, Department of Psychosomatic Medicine and Psychotherapy**

Date **03-Feb-2024**

COI **no competing interests**

Thank you for the opportunity to review Manuscript ID bmjopen-2023-082498, entitled "Living with Uncertainty: a qualitative interview study in primary sclerosing cholangitis (PSC)" for BMJ Open.

This is an important and comprehensive paper highlighting the experience of people with a rare liver condition. The qualitative approach is appropriate and provides meaningful findings. I recommend this paper is published, however, some major issues need to be addressed before.

General comments:

- I generally recommend to use the expression "patients with PSC" instead of "PSC patients".

- There are some minor spelling mistakes, missing words etc., so please check the manuscript accordingly (f.e. Abstract, page 3, line 47)

- In some places, the paper refers to the corona pandemic. As the data was collected long before the pandemic and it has no scientific relevance for the paper, I suggest deleting all references to the pandemic from the paper.

Title:

- Please add "patients with" to the title.

- The title could also be formulated more broadly; it currently refers to a very specific result, even though there were many other interesting topics found in the data; the theme of the journey is not well illustrated by this title

Abstract:

- I suggest to delete the last sentences of the conclusions, since it repeats the first sentence of the objectives.

- A suggestion, present the result in the same way and order in the abstract as in the text- i.e. re-ferring to the structure of the “journey”.

Strenghts and limitations:

- Please organize the section by strengths and limitations, they are currently mixed up.

- Please put point 1 into perspective, as this is not the first and only study to look qualitatively at the patient experience in patients with PSC.

Introduction:

- The introduction should be elaborated, the authors mention some predecessor studies without naming their main results in terms of content. In particular, the recently published study by Lö-sken et al. (2023) is very similar to this study in terms of methodology and content. It should become clear what the current study can further contribute to these already existing results, what is the added value, and how can the current study be embedded in this context.

- I would also like to learn about a theoretical framing of the research question in the introduction part.

Methods:

- The qualitative approach and especially the research paradigm and guiding theory need elaboration. Please see standards for reporting of qualitative research (SRQR), page 8.

- Please also elaborate on potential or actual interaction between researchers' characteristics and the research question, approach, methods, results, and/or transferability (SRQR, page 3).

- Study design and sampling: I suggest to also elaborate in which scientific context this study was conducted, and what was the underlying research interest and question, especially since the collection of the data was a long time back, please comment.

- Data collection: What criteria were used to compile the topic guide for the interviews, based on which scientific theory and research questions?

- Interview analysis; The authors must please elaborate the steps taken in analyzing the data thematically. How was the data coded, by how many coders, by the help of any software, by

discussion in a team, etc. The here mentioned reference by Braun and Clarke is outdated. Readers should be able to understand and follow the procedure of data analysis.

Results:

- My main criticism of the result section is the quantitative approach used in some aspects (stage 1 symptoms, figure 2, counting quotes of patients), quantification of qualitative data should always be avoided and does not correspond to the logic of qualitative data evaluation. I therefore recommend to rewrite these parts of the results section.

- It should also be elaborated how the main themes and sub-themes were identified, did the authors establish a bottom-up or top-down approach?

- Suggestion: Move the last paragraph of “loss in faith in healthcare to “isolation and peer support”, this seems more coherent in terms of content.

- Minor questions: What were the screening questions that patients answered before the inter-views? How was severity of disease assessed?

Discussion:

- The discussion is interesting, however, the authors could try to better embed their findings in the theoretical literature on health and illness.

- At this point, please also refer your own results to the results of previous similar studies, e.g. work out similarities and differences (Lösken et al., 2023)

- In terms of clinical implications, the manuscript would be improved if the authors could sug-gest more tangible ways in which health care practitioners can better support people with PSC. Can they give more specific types of intervention to promote acknowledgement of the patient experience, awareness raising, ‘educating’ patients and health care providers.

References:

- Many of the cited references are outdated, I suggest a search for current relevant literature.

I hope my comments and suggestions are helpful to improve the paper.

VERSION 1 - AUTHOR RESPONSE

Reviewer: 1

Dr. Veronica Ranieri, Tavistock and Portman NHS Foundation Trust, University College London

Reviewer 1’s comment:

Though there are fewer published studies on the lived experiences and psychological impact of PSC (of which the authors describe in their results) in comparison to those chronicling the physical symptoms and medical management of the illness, I would suggest that the authors provide a more ample

introduction and comparison to previous literature within the discussion on what is already known and how their study's findings differ.

Authors' response: Thank you for your helpful comments. Text has been added to reflect the above suggestion.

Reviewer 1's comment: 'The end of the road' may reflect the anxiety and hopelessness some individuals feel as they think of or approach advanced liver illness. I wonder, however, if this could be revised as am mindful that some individuals with PSC may read this and feel worse.

Authors' response: Thank you for your suggestion. Titles have been adjusted in the text and within the figure.

Reviewer 1's comment: I would be cautious about including the statement that "women can have stronger feelings of vulnerability to illness, greater felt stress and an overall different perception of their own health when compared to men". This may or may not be true, depending on whether you believe that women experience these difficulties more or are just more likely to report these.

Authors' response: Thank you for your advice - wording has been revised accordingly.

Reviewer: 2

Dr. Anne Toussaint, University Medical Center Hamburg-Eppendorf

General comments:

Reviewer 2's comment: I generally recommend to use the expression "patients with PSC" instead of "PSC patients". There are some minor spelling mistakes, missing words etc., so please check the manuscript accordingly (f.e. Abstract, page 3, line 47).

Authors' response: Thankyou – wording has been changed as advised to "patients with PSC" and further proof-reading completed to identify any errors in spelling.

Reviewer 2's comment: In some places, the paper refers to the corona pandemic. As the data was collected long before the pandemic and it has no scientific relevance for the paper, I suggest deleting all references to the pandemic from the paper.

Authors' response: Many thanks – most references removed as recommended. However, one mention of the pandemic is retained within the Discussion; this was felt relevant by the authors to the current residual reliance on remote telemedical consultations and the potential impact of these on access to specialist care in PSC and the complex patient-doctor dynamics involved.

Title:

Reviewer 2's comment: Please add "patients with" to the title.
The title could also be formulated more broadly; it currently refers to a very specific result, even though there were many other interesting topics found in the data; the theme of the journey is not well illustrated by this title.

Authors' response: Thankyou – title amended as advised

Reviewer 2's comment: I suggest to delete the last sentences of the conclusions, since it repeats the first sentence of the objectives.
A suggestion, present the result in the same way and order in the abstract as in the text- i.e. re-ferring to the structure of the "journey".

Authors' response : Graciously received. Content has been revised based on the above.

Strenghts and limitations:

Reviewer 2’s comment: Please organize the section by strengths and limitations, they are currently mixed up. Please put point 1 into perspective, as this is not the first and only study to look qualitatively at the patient experience in patients with PSC.

Authors’ response: Thank you for your valued input – content has been revised based on the above.

Reviewer 2’s comment: The introduction should be elaborated, the authors mention some predecessor studies without naming their main results in terms of content. In particular, the recently published study by Lö-sken et al. (2023) is very similar to this study in terms of methodology and content. It should become clear what the current study can further contribute to these already existing results, what is the added value, and how can the current study be embedded in this context.

Authors’ response: Many thanks – further background on other research in this area, including the 2023 Losken study, has been added into the manuscript as advised.

Reviewer 2’s comment: The qualitative approach and especially the research paradigm and guiding theory need elaboration. Please see standards for reporting of qualitative research (SRQR), page 8.

Authors’ response: Many thanks – further elaboration as requested has been incorporated into the methods section of the manuscript.

Reviewer 2’s comment: Please also elaborate on potential or actual interaction between researchers’ characteristics and the research question, approach, methods, results, and/or transferability (SRQR, page 3).

Authors’ response: Many thanks – further elaboration as requested has been incorporated into the methods section of the manuscript.

Reviewer 2’s comment: Study design and sampling: I suggest to also elaborate in which scientific context this study was conducted, and what was the underlying research interest and question, especially since the collection of the data was a long time back, please comment. Data collection: What criteria were used to compile the topic guide for the interviews, based on which scientific theory and research questions?

Authors’ response: Many thanks – further elaboration as requested has been incorporated into the methods section of the manuscript.

Reviewer 2’s comment: Interview analysis; The authors must please elaborate the steps taken in analyzing the data thematically. How was the data coded, by how many coders, by the help of any software, by discussion in a team, etc. The here mentioned reference by Braun and Clarke is outdated. Readers should be able to understand and follow the procedure of data analysis.

Authors’ response: Many thanks – further elaboration as requested has been incorporated into the methods section of the manuscript.

Reviewer 2’s comment: My main criticism of the result section is the quantitative approach used in some aspects (stage 1 symptoms, figure 2, counting quotes of patients), quantification of qualitative data should always be avoided and does not correspond to the logic of qualitative data evaluation. I therefore recommend to rewrite these parts of the results section.

Many thanks – this section has been modified to exclude quantification as much as possible The authors do however suggest that some indication of the frequency of the themes identified is helpful in table 1 & figure 2, and may make the article more accessible, especially to readers who may be more familiar with traditional qualitative research methods.

Reviewer 2’s comment: Suggestion: Move the last paragraph of “loss in faith in healthcare to “isolation and peer support”, this seems more coherent in terms of content.

Authors’ response: Many thanks – paragraph moved as suggested

Reviewer 2’s comment: Minor questions: What were the screening questions that patients answered before the inter-views? How was severity of disease assessed?

Authors’ response: Many thanks – elaboration on both points has been added into the results section.

Reviewer 2’s comment: The discussion is interesting, however, the authors could try to better embed their findings in the theoretical literature on health and illness. At this point, please also refer your own results to the results of previous similar studies, e.g. work out similarities and differences (Lösken et al., 2023)

Authors’ response: Many thanks – further elaboration as requested has been incorporated into the discussion section of the manuscript.

Reviewer 2’s comment: In terms of clinical implications, the manuscript would be improved if the authors could sug-gest more tangible ways in which health care practitioners can better support people with PSC. Can they give more specific types of intervention to promote acknowledgement of the patient experience, awareness raising, ‘educating’ patients and health care providers.

Authors’ response: Thank you for your input – adjustments have been made to better reflect the types of intervention available to patients living with PSC.

Reviewer 2’s comment: Many of the cited references are outdated, I suggest a search for current relevant literature. I hope my comments and suggestions are helpful to improve the paper.

Authors’ response: Gratefully received- references have been reviewed.

VERSION 2 - REVIEW

**Reviewer**	**1**
**Name**	**Ranieri, Veronica**
**Affiliation**	**Tavistock and Portman NHS Foundation Trust, Research & Development**
**Date**	**09-Nov-2024**
**COI**	

The authors have addressed most changes. I think they could have cited more papers on the topic to contextualise their findings (eg https://pscsupport.org.uk/research-weve-funded/), however, it is a good paper!

**Reviewer**	**2**

Name Toussaint, Anne

Affiliation University Medical Center Hamburg-Eppendorf, Department of Psychosomatic Medicine and Psychotherapy

Date 15-Nov-2024

COI

Thank you very much for revising the manuscript. The authors have implemented all my suggestions in the manuscript to my satisfaction, so that I recommend it for publication in BMJ Open.

Just two general comments, from a reviewer's point of view it would be desirable to present the changes and the rationale for them in the response letter, and to indicate the changed text passages with page and line numbers, the response letter should be able to stand on its own. In addition, the comments of the conversation of the author group should usually be removed from the manuscript with tracked changes before submission.

Translated with DeepL.com (free version)

VERSION 2 - AUTHOR RESPONSE

Reviewer 1: Dr. Veronica Ranieri, Tavistock and Portman NHS Foundation Trust, University College London

1. The authors have addressed most changes. I think they could have cited more papers on the topic to contextualise their findings (eg https://pscsupport.org.uk/research-weve-funded/), however, it is a good paper!

   Thank you for your comments. The referenced papers have been meticulously reviewed to ensure we have added the context.

Reviewer: 2
Dr. Anne Toussaint, University Medical Center Hamburg-Eppendorf

1. Thank you very much for revising the manuscript. The authors have implemented all my suggestions in the manuscript to my satisfaction, so that I recommend it for publication in BMJ Open.

2. Just two general comments, from a reviewer's point of view it would be desirable to present the changes and the rationale for them in the response letter, and to indicate the changed text passages with page and line numbers, the response letter should be able to

stand on its own. In addition, the comments of the conversation of the author group should usually be removed from the manuscript with tracked changes before submission.

Thank you for all of your comments to assist in preparing this paper for publication. For this round of comments, we have added the changes and line numbers. We will be sure to include these in future submissions. It has been indicated by the journal that the authorship statement must remain in the document in this case, but we will ensure to check the requirements in future journals and submissions.
